# Tribo-synergism in titanium complex grease using micro and nano particles

**DOI:** 10.1371/journal.pone.0323444

**Published:** 2025-05-12

**Authors:** Ming Niu, Yunbo Gao, Xinyu Wang, Zhenghu Liu

**Affiliations:** 1 School of Traffic and Vehicle Engineering, Wuxi University, Wuxi, Jiangsu, China; 2 SPIC Gansu Electric Power Co., Ltd, Lanzhou, Gansu, China; Bina Nusantara University, INDONESIA

## Abstract

Micro-nano additive-enhanced lubricating greases are pivotal for extreme-condition tribology, yet optimizing synergistic additive concentrations remains constrained by conventional experimental designs. This study employs a central composite design (CCD) coupled with MATLAB response surface methodology to precisely determine optimal concentrations of nano-graphite (N-G), graphene (GN), and potassium borate (PB) in titanium complex grease. Fifteen formulations were tested under progressive loads (98–598 N) via four-ball tribometry, with SEM/XPS characterizing wear mechanisms. The synergistic grease (G-MX: 0.83 wt% N-G, 0.05 wt% GN, 2.59 wt% PB) reduced the average friction coefficient by 45.3% and wear scar diameter by 23.3% versus base grease, surpassing single-additive variants. The CCD-MATLAB framework addressed sampling limitations of prior orthogonal methods, enabling optimization beyond discrete testing points. Mechanistic analysis revealed a dual lubrication regime: physically adsorbed films (soap molecules and refined PB particles) dominated at low loads, while chemically bonded tribofilms (Fe₃C, B₂O₃, TiO₂) ensured wear resistance under extreme pressures.

## 1. Introduction

Lubricating greases are crucial for reducing friction, wear, energy consumption, and extending the lifespan of machines. The use of micro and nano particles in improving the tribological properties of grease has garnered significant attention in recent times [1–4].

Since graphene was discovered in 2004, experts and scholars have conducted extensive research on graphene nanomaterials [5]. Graphene (GN) has attracted considerable attention due to its unique structure and remarkable tribological properties [6–9]. As a kind of additives, it has been applied into lubricating oil [10,11] and lubricating grease [12,13], and the mechanism has been uncovered as the formation of deposited film and the chemical film. As a new type of additive, GN has great potential for application.

Different from a single layer of graphene, nano-graphite consists of multiple layers of carbon atoms with nanoscale thickness. Nano-graphite has been shown to improve the tribological performance of lubricants [14]. It has also been proven to be an excellent additive in lubricating oils for enhancing tribological properties [15–18]. Moreover, research has shown that nano-graphite-modified lubricating greases exhibit better tribological properties than base greases [19,20].

Borate additives are receiving increasing attention as excellent additives due to their advantages of good extreme pressure and anti-wear properties, as well as being non-toxic and odorless. Various borate additives, such as cerium borate [21,22], magnesium borate [23], lanthanum borate [24], calcium borate [25–27], barium borate [28], titanium borate [29], and zinc borate [30], have proven to possess good extreme pressure and anti-wear properties. Li et al. [31] disclosed that potassium borate particles can generate lubricating films containing Fe₂O₃, Fe_x_Bγ, and B₂O₃ in boundary lubrication. With excellent load-bearing capacity and lubricating properties, potassium borate is a promising additive for applications.

Although individual additives have been found to enhance the tribological properties of lubricants [20,32–36], combining multiple types of particles has been shown to result in even greater improvements [37–40]. The great improvements of tribological properties are attributed to the complementary roles of the physical and chemical films created by the components of additives in the boundary lubrication [41,42]. The key to the combination is the determination of the optimal concentration of each additive.

To determine the optimal concentration combinations, the following studies were conducted. Alghani [43] designed a six-sample experiment and identified the combination of 0.4 wt% TiO₂ and 0.2 wt% graphene as the optimal mixture to improve the tribological behavior of base oil. Xin [37] selected the optimal concentration combinations of antimony dialkyldithiocarbamate (SbDTc), zinc dialkyldithiophosphate (ZDDP), and sulfurized isobutylene (SE) to enhance the extreme pressure properties of lubricating greases. The study evaluated 10 different formulas using the three-component simplex-centroid design method. Muhammad [44] optimized the tribological performance of engine oil using BN/Al₂O₃ nanoparticles. Based on the 9-sample orthogonal method, the optimal combination was determined to be 0.05 vol.% Al₂O₃ and 0.5 vol.% hBN. Although these studies determined the optimal formulations, the insufficient number of experimental samples makes it difficult to fully reflect the tribological law. Moreover, the optimal formulations were directly derived from the experimental results, without subsequent optimization processes to improve accuracy.

Initial evidence indicates that the application of graphene, nano-graphite, and potassium borate particles as lubricant additives holds considerable potential. Specifically, nano-graphite (N-G) has been proven to reduce friction through interlayer sliding [14,20], graphene (GN) forms a robust boundary film due to its high mechanical strength [43,45], and potassium borate (PB) generates a chemically reactive B₂O₃ layer under extreme conditions [46,47]. Their combination is hypothesized to synergistically enhance both physical and chemical lubrication mechanisms. Therefore, this study focuses on determining the optimal combinations of nano-graphite, graphene, and potassium borate additive concentrations, with the goal of maximizing the tribological performance of titanium complex grease. By fitting the tribological experimental results with MATLAB, refined optimal formulations unconstrained by experimental limitations were obtained. The friction test adopts a compound experimental design involving 15 grease samples, assigning five concentration levels to each additive particle, which achieves an optimal balance between experimental cost-efficiency and sampling comprehensiveness. The worn surfaces were characterized using SEM and XPS in an attempt to interpret the boundary lubrication mechanism of synergistically modified titanium complex grease.

## 2. Materials and methods

### 2.1. Materials

The laboratory prepared the complex grease used in this study. The grease consists of complex titanium soap, mineral base oil, and additives. The thickener was prepared using stearic acid, benzoic acid, tetraeisopropyl titanate, and distilled water as the main materials.

**Fig 1:**
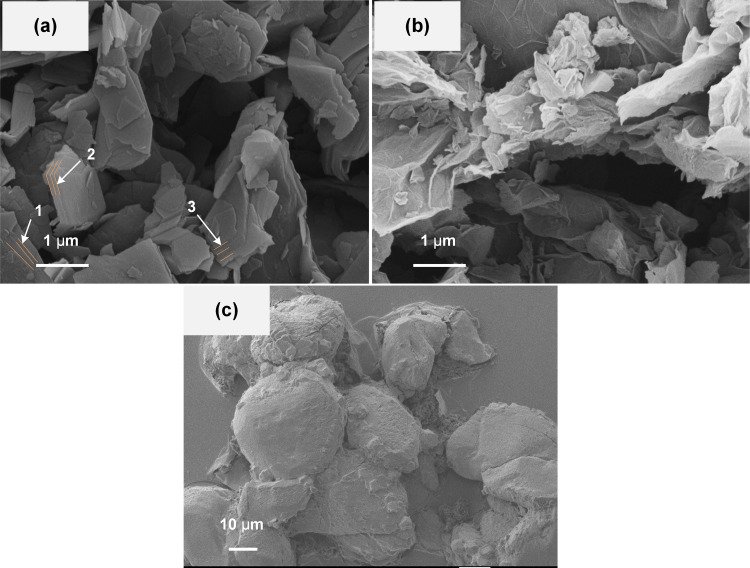
SEM morphologies of friction reducing and antiwear additives. (a) N-G, (b) GN, (c) PB.

In this study, the friction reducing and antiwear additives used are nano graphite (NG), graphene (GN), and micro potassium borate (PB). The SEM morphologies of these additives are shown in Fig 1. Fig 1a shows that N-G is stacked by thin layers with a thickness of approximately 20–40 nm, as marked by arrows. GN (shown in Fig 1b) consists of curly layers that entangle one another. PB (shown in Fig 1c), on the other hand, exhibits a massive particle morphology with a size of about 30 μm.

### 2.2. Experimental equipment and test methods

#### 2.2.1. Tribological modification of titanium complex grease.

In this study, the titanium complex grease was modified with N-G, GN, and PB additives. The dispersion of additives in the base grease was achieved using an ultrasonic cleaner (Type: SB 2200) and was added to the reaction mixture at the cooling stage at the required weight concentrations. The modified greases were then ground three times. The optimum single weight concentration of N-G, GN, and PB was determined to be 0.8 wt%, 0.06 wt%, and 3.0 wt%, respectively, based on previous studies. For clarity, the base grease and each modified titanium complex grease were named and numbered sequentially in Table 1.

**Table 1:** Serial numbers of base and modified titanium complex greases.

Additives	Additive concentrations (wt %)	Names of greases	Serial numbers of greases
--	--	Base grease	G
N-G	0.8	N-G modified grease	G-MA
GN	0.06	GN modified grease	G-MB
PB	3.0	PB modified grease	G-MC
N-G + GN + PB	Optimum synergic concentrations (Determine in Part 3.1.1)	Synergically modified grease	G-MX

#### 2.2.2. Solving method of optimum synergic concentrations of three kinds of additives.

**Fig 2:**
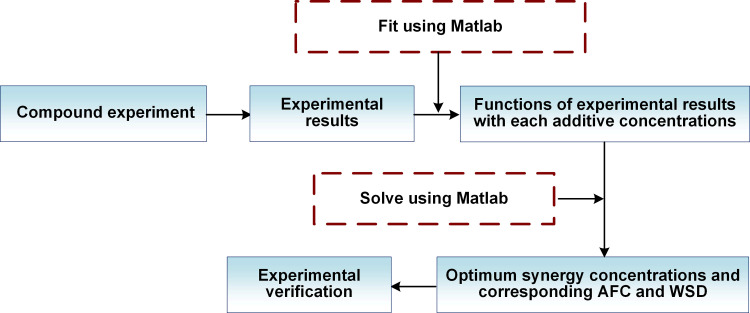
Schematic diagram of compound experiment-responding surface method.

**Fig 3:**
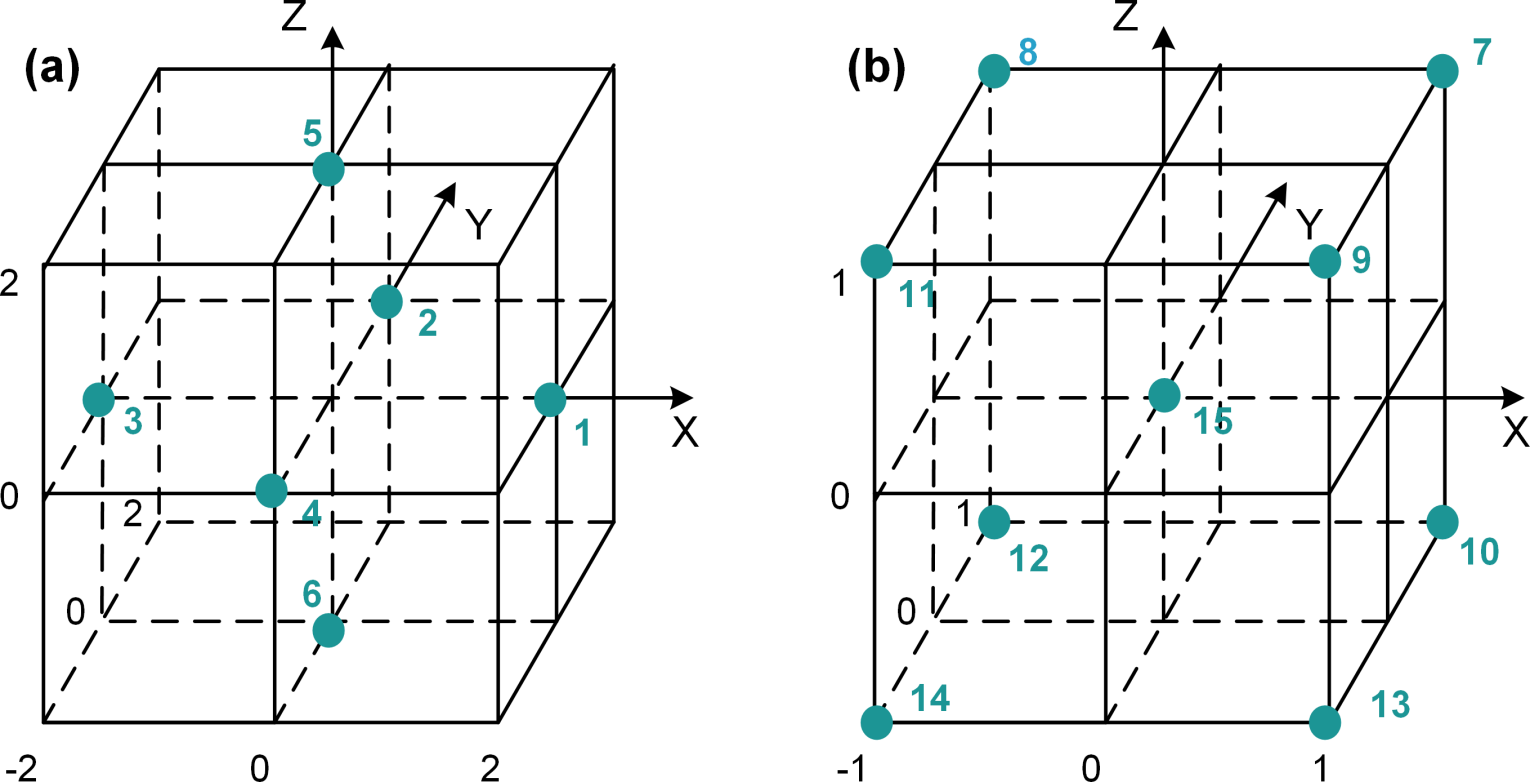
Diagram for the point choosing in compound experiment designed. (a) cubic grids A, (b) cubic grids B.

This study employed a combination of compound experiment and MATLAB mathematical fitting to identify the optimal synergistic concentrations of N-G, GN, and PB. The resulting diagram is displayed in Fig 2. The central composite design (CCD) [48], employing 5 levels and 3 factors, was selected for its efficiency in exploring quadratic responses with only 16 experimental runs. This design includes factorial points (7–14), facial points (1–6), and center points (15), enabling a comprehensive analysis of interactions between additives (presented in Fig 3). The practical concentrations of each additive at these levels are provided in Table 2. The most representative additive formulation was chosen based on the results of this analysis.

**Table 2:** Correspondence between level and experimental values of the variables studied.

Iterm	Representation	Values				
Levels values	(X, Y, Z)	-2	-1	0	1	2
Practical concentrations for N-G (wt%)	x	0.4	0.6	0.8	1.0	1.2
Practical concentrations for GN (wt%)	y	0.02	0.04	0.06	0.08	0.10
Practical concentrations for PB (wt%)	z	1	2	3	4	5

Tribological experiments were subsequently conducted on the 15 formulated titanium complex greases. Precise mathematical equations relating the concentrations of each additive to AFC and WSD were established through MATLAB curve fitting analysis. This computational process enabled the determination of critical extreme points within the derived equations. Through systematic optimization between these identified extremum points, the synergistic concentration combination of the three additives was ultimately determined, representing the optimal balance between friction reduction and wear resistance performance.

#### 2.2.3. Tribological properties tests.

The tribological properties of greases were analyzed using the four-ball tester (Type: MRS-10) and the worn scars of the upper ball were examined. Prior to testing, GCr15 bearing steel balls (12.7 mm diameter, RC 59–61 hardness) underwent standardized preparation involving ultrasonic cleaning (SB2200 model) in acetone for 30 minutes followed by oven drying.The friction reducing properties of the greases were evaluated by average AFC, and anti-wear properties by WSD of steel balls. The tribological properties of fresh greases were compared with all experimental conditions verified through triplicate tests to ensure data reproducibility before final averaging.

(1) Composite performance evaluation

The average friction coefficient (AFC) and wear scar diameter (WSD) of each grease/ball system were determined using the SH/T 0204–92 standard method. Testing parameters included a constant load of 392 N and rotational speed of 1450 rpm maintained for 1 hour.

(2) Boundary lubrication assessment

A progressive loading protocol (98 N, 206 N, 304 N, 398 N, 510 N, 598 N) was implemented to evaluate five grease formulations including (Grease G-MA, G-MB, and G-MC), and synergistic grease (Grease G-MX). All tests were performed at 1450 rpm with 1-hour duration per load stage, with AFC and WSD measurements recorded systematically.

#### 2.2.4. Analysis of the worn surface.

Post-tribological testing, comprehensive wear scar analysis was conducted on steel balls lubricated with: base grease (Grease G), individual additive-modified greases (G-MA, G-MB, G-MC), and the synergistic formulation (G-MX). Specimen preparation involved sequential solvent cleaning protocols - initial degreasing with 95% acetone (20 min ultrasonic immersion, SB2200 system) followed by ethanol rinsing (10 min) to remove residual contaminants, concluding with drying.

The worn surface was characterized using SEM (Type: Merlin Compact, made in Germany) and XPS (Type: ESCALAB 250Xi, made in America). The XPS analysis used Al-Kα radiation (λ = 0.8339 nm, 1486.6 eV) with the binding energy of contaminated carbon (C1s: 284.80 eV) as the reference, with a measurement accuracy of approximately ± 0.3 eV. The high-resolution XPS spectra were fitted using XPS PEAK 4.1 software, programmed with Gaussian-Lorentzian.

## 3. Results

### 3.1. Synergy modification for titanium complex grease

#### 3.1.1. Determination of optimum synergy concentrations of each additive.

The compound experiments and corresponding results for AFC and WSD of modified greases are presented in Table 3. The quadratic regression model was chosen to account for potential nonlinear interactions between additives. The high R² values (0.9835 for AFC and 0.9665 for WSD) confirmed the model’s accuracy in predicting tribological responses beyond the tested points.

**Table 3:** Compound experimental design and corresponding results (392N, 1450rpm, 1 h).

No.	Level (X, Y, Z)	*x* (N-G (wt%))	*y* (GN (wt%))	*z* (PB (wt%))	AFC	WSD (μm)
1	(2, 0, 0)	1.2	0.06	3	0.068	596
2	(0, 2, 0)	0.8	0.1	3	0.069	597
3	(-2, 0, 0)	0.4	0.06	3	0.063	583
4	(0, -2, 0)	0.8	0.02	3	0.061	581
5	(0, 0, 2)	0.8	0.06	5	0.070	598
6	(0, 0, -2)	0.8	0.06	1	0.062	582
7	(1, 1, 1)	1.0	0.08	4	0.076	614
8	(-1, 1, 1)	0.6	0.08	4	0.063	584
9	(1, -1, 1)	1.0	0.04	4	0.064	585
10	(1, 1, -1)	1.0	0.08	2	0.062	583
11	(-1, -1, 1)	0.6	0.04	4	0.059	576
12	(-1, 1, -1)	0.6	0.08	2	0.058	575
13	(1, -1, -1)	1.0	0.04	2	0.057	574
14	(-1, -1, -1)	0.6	0.04	2	0.067	580
15	(0, 0, 0)	0.8	0.06	3	0.058	576


$$AFC=0.1972-0.1560x-1.3750y-0.0296z+0.6875xy+0.0150xz+0.1250yz+0.0479x^{2}+4.4792y^{2}+0.0020z^{2}$$ (1)

$$WSD=847.7917-303.0208x-2809.3750y-58.3125z+1406.2500xy+29.3750xz+268.7500yz+92.7083x^{2}+8958.3333y^{2}+3.8333z^{2}$$ (2)

Table 4 presents the critical minima derived from Equations (1, 2), corresponding to dual optimization parameters for achieving minimum AFCmin and WSDmin. Through statistical reconciliation of these paired parameter sets, the synergistic formulation was established with optimal constituent concentrations: 0.8307 wt% N-G, 0.0531 wt% GN, and 2.5897 wt% PB, demonstrating balanced tribological enhancement through additive interaction.

**Table 4:** Fitted minimum points and the optimum synergy concentration of each additive.

Item	x (N-G (wt%))	y (GN (wt%))	z (PB (wt%))
AFCmin (0.0568)	0.8483	0.0523	2.5832
WSDmin (572.99 μm)	0.8131	0.0540	2.5963
Synergistic concentration	0.8307	0.0531	2.5897

#### 3.1.2. Analysis of the response surfaces.

The ternary equations (Eqs.1, 2) resist 3D surface representation due to mathematical complexity. We addressed this by fixing individual additives at optimized concentrations (from prior determination) through parametric analysis, generating six bivariate equations (Eqs.3–8) through pairwise component variation. This approach enabled multidimensional tribological relationship visualization and systematic evaluation. For example, in the AFC_*xy*_ model, the variable *z* is set to 2.5897 to simplify the analysis, and thus only the variables *x*, *y*, *xy*, *xx*, and *yy* are included. The concentration constant was maintained accordingly to ensure consistency in the model formulation.


$${AFC}_{xy}=0.0479x^{2}+0.6875xy\;–\;0.1173x\;+4.4792y^{2}-1.0521y+0.1341$$ (3)

$${AFC}_{xz}=\;0.0479x^{2}+0.015xz\;–0.1200x+0.002z^{2}-0.0231z+0.1375$$ (4)

$${AFC}_{yz}=\;4.4792y^{2}+\;0.152yz\;–0.7918y+0.002z^{2}-\;0.0169z+0.0993$$ (5)

$${WSD}_{xy}=92.708x^{2}+1406.3xy-226.7545+8958.3y^{2}-2111.6y+722.23$$ (6)

$${WSD}_{xz}=92.708x^{2}+29.375xz-227.083x+3.833z^{2}-43.8z+722.2079$$ (7)

$${WSD}_{yz}=8958.3y^{2}+268.75yz-1666y+3.8333z^{2}-34.427z+662.697$$ (8)

**Fig 4:**
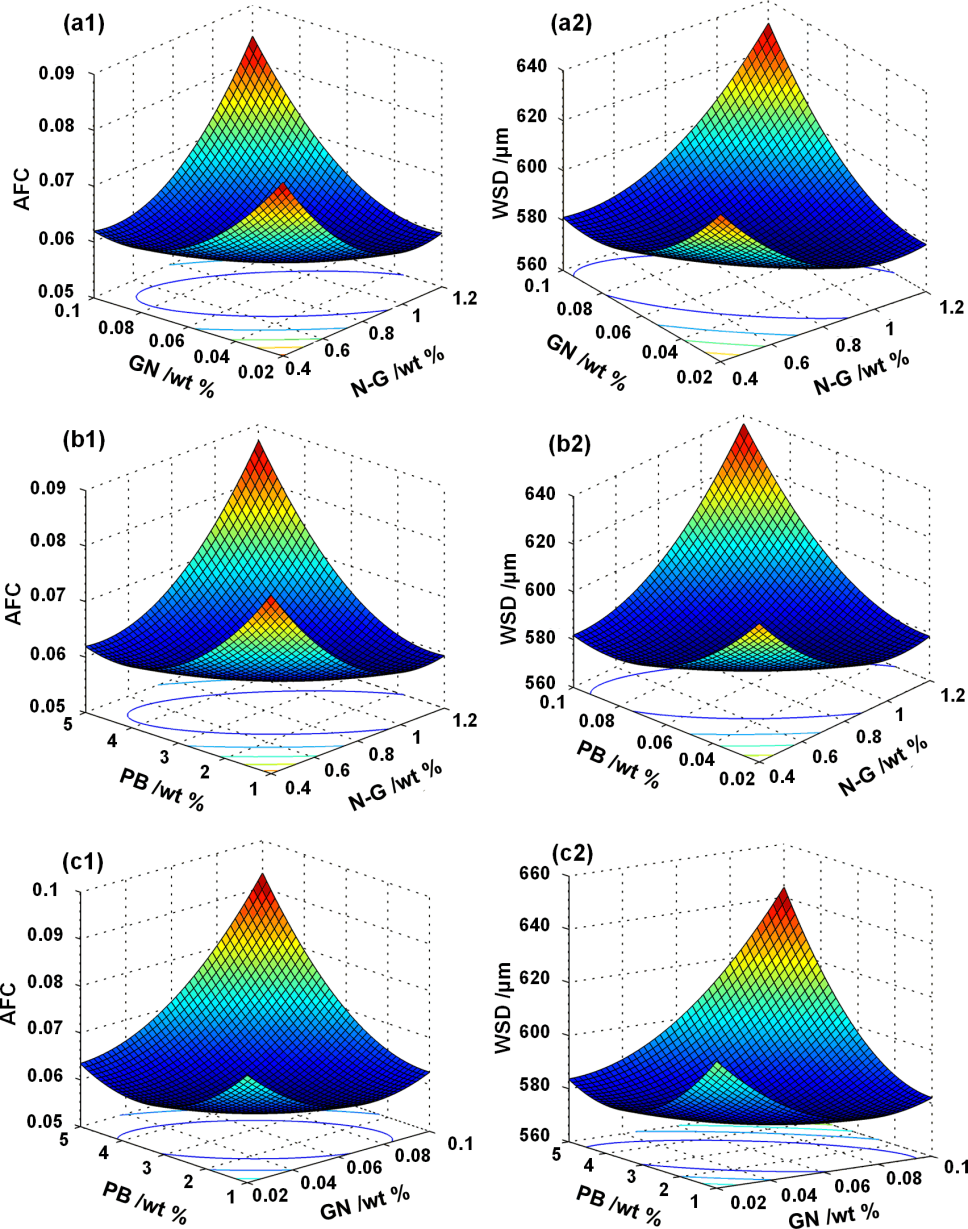
Response surfaces of AFC ((a1), (b1), (c1)) and WSD ((a2), (b2), (c2)) as functions of the concentrations of either two additives. (a1) and (a2) N-G and GN (the concentration of PB 2.5897 wt%), (b1) and (b2) N-G and PB (the concentration of GN 0.0531 wt%), (c1) and (c2) GN and PB (the concentration of N-G 0.8307 wt%).

Employing Eqs.3–5, we constructed predictive response surface models to systematically correlate the Anti-Friction Coefficient (AFC) with individual additive concentrations within the tribological system. Fig 4(a1)–(c1) demonstrate a critical concentration threshold for each component: AFC exhibited concentration-dependent reduction within optimal ranges (N-G: 0.4–0.85 wt%, GN: 0.02–0.053 wt%, PB: 1–2.59 wt%), beyond which friction coefficients rebounded. Parallel analysis of WSD in Fig 4(a2)–(c2) revealed analogous nonlinear trends, with wear resistance first improving then deteriorating at near-identical critical concentrations (N-G: 0.82 wt%, GN: 0.054 wt%, PB: 2.59 wt%). This biphasic behavior aligns with Singh’s lubrication-agglomeration theory [49], where optimal additive dosing enhances tribofilm formation while excessive loading induces particle aggregation and grease microstructure disruption.

To test the model’s accuracy, we performed experimental tests on the optimized grease using a four-ball tester. Table 5 compares measured AFC and WSD values with model predictions, showing minimal deviations of 0.42% (AFC) and 0.53% (WSD). These results confirm both the accuracy of the optimized additive concentrations and the validity of the concentration-response models for friction and wear behavior.

**Table 5:** Comparation of tested and fitted results of AFC and WSD for Grease G-MX.

Item	Fitted values	Tested values	Relative errors (%)
AFC	0.0568	0.0571	0.42
WSD (μm)	572.99	570.62	0.53

### 3.2. Comparison of tribological properties

**Fig 5:**
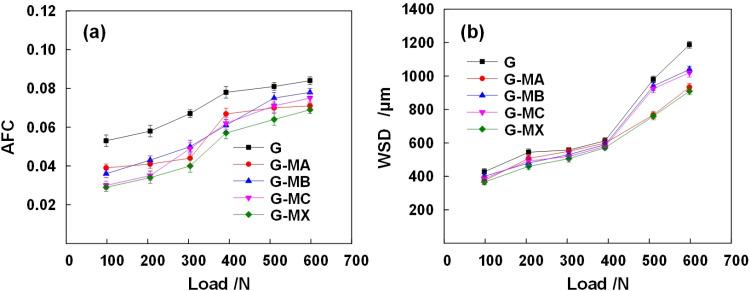
Comparation of variations of AFC and WSD with applied loads lubricated by each grease sample. (a) The relationship of AFC and applied loads, (b) The relationship of WSD and applied loads.

To comprehensively evaluate the tribological performance of the Synergistic-grease (G-MX), the four-ball test results were analyzed across six applied loads (98–598 N) and compared to base grease (G) and single-additive modified greases (G-MA, G-MB, G-MC) (Fig 5). Key findings include:

#### 3.2.1. Friction reduction.

Under the boundary lubrication regime, the Synergistic-grease exhibited a 17.9–45.3% reduction in average friction coefficient (AFC) compared to base grease (Fig 5a and Table 6). Especially before 392 N, G-MX achieved an AFC ranging from 0.029 to 0.04, outperforming base grease (0.053–0.067), G-MA (0.039–0.044), G-MB (0.036–0.04), and G-MC (0.03–0.049) (Fig 5a and Table 6). Specifically, it decreased by 40.3–45.3% compared to base grease, by 10.0–34.5% compared to G-MA, by 24.1–26.4% compared to G-MB, and by 2.9–22.5% compared to G-MC (Table 6)

**Table 6:** Detailed Information of average frictional coefficient (AFC) and Reduction Efficiency of All Grease Samples Under Various Loads.

load	AFC					Reduction (%)			
	G	G-MA	G-MB	G-MC	G-MX	G	G-MA	G-MB	G-MC
98	0.053	0.039	0.036	0.03	0.029	45.3	34.5	24.1	3.4
206	0.058	0.041	0.043	0.035	0.034	41.4	20.6	26.4	2.9
304	0.067	0.044	0.05	0.049	0.04	40.30	10.0	25.0	22.5
392	0.078	0.067	0.061	0.062	0.057	26.9	17.5	7.0	8.8
510	0.081	0.07	0.075	0.071	0.064	20.99	9.4	17.2	10.9
598	0.084	0.071	0.078	0.075	0.069	17.9	2.9	13.0	8.7

#### 3.2.2. Anti-wear performance.

Under the boundary lubrication regime, WSD for G-MX ranged from 366–910 μm, significantly lower than those of the base grease (427–1187 μm) and single-additive greases (e.g., G-MA: 374–933 μm; G-MB: 400–1040 μm; G-MC: 386–1020 μm) (Fig 5b). Notably, under loads exceeding 392 N, G-MX reduced WSD highly by 22.4–23% compared to the base grease. When compared to single-additive modified greases (G-MA, G-MB, G-MC), G-MX further demonstrated reductions in WSD of 0.9–2.5%, 14.3–23.7%, and 12.1–21.8%, respectively (Fig 5b and Table 7).

**Table 7:** Detailed Information of Wear Scar Diameter (WSD) and Reduction Efficiency of All Grease Samples Under Various Loads.

load	WSD (μm)					Reduction (%)			
	G	G-MA	G-MB	G-MC	G-MX	G	G-MA	G-MB	G-MC
98	427	374	400	386	366	14.3	2.2	9.3	5.5
206	544	507	480	492	460	15.4	10.2	4.4	6.7
304	558	550	530	519	506	9.32	08.7	4.7	2.6
392	612	598	589	578	571	6.8	4.8	3.2	1.3
510	980	767	940	925	760	22.4	0.9	23.7	21.8
598	1187	933	1040	1020	910	23.3	2.5	14.3	12.1

#### 3.2.3. Insights into boundary lubrication.

The AFC and WSD of Synergistic-grease outperformed the base grease and the single-modified grease across all loads, and the load-dependent performance improvements highlight the importance of additive synergism in maintaining effective lubrication films. The AFC and WSD values of all grease formulations progressively increased with elevated applied loads, reflecting a corresponding deterioration in the anti-wear and friction-reduction performance. This trend can be attributed to the boundary lubrication regime, where the protective boundary film becomes increasingly prone to detachment under higher contact pressures. The superior AFC and WSD of Synergistic-grease compared to other greases can be attributed to the improvement of boundary lubrication, which results from the formation of more effective protective films due to the synergistic additives.

### 3.3. Analysis on the worn surface

#### 3.3.1. SEM analysis on the worn surface.

To elucidate the lubrication mechanisms of titanium complex grease, we analyzed the wear scar morphology of steel balls lubricated with Grease G, G-MA, G-MB, G-MC, and G-MX under a 392 N load at 1450 rpm for 60 minutes using scanning electron microscopy (SEM).

**Fig 6:**
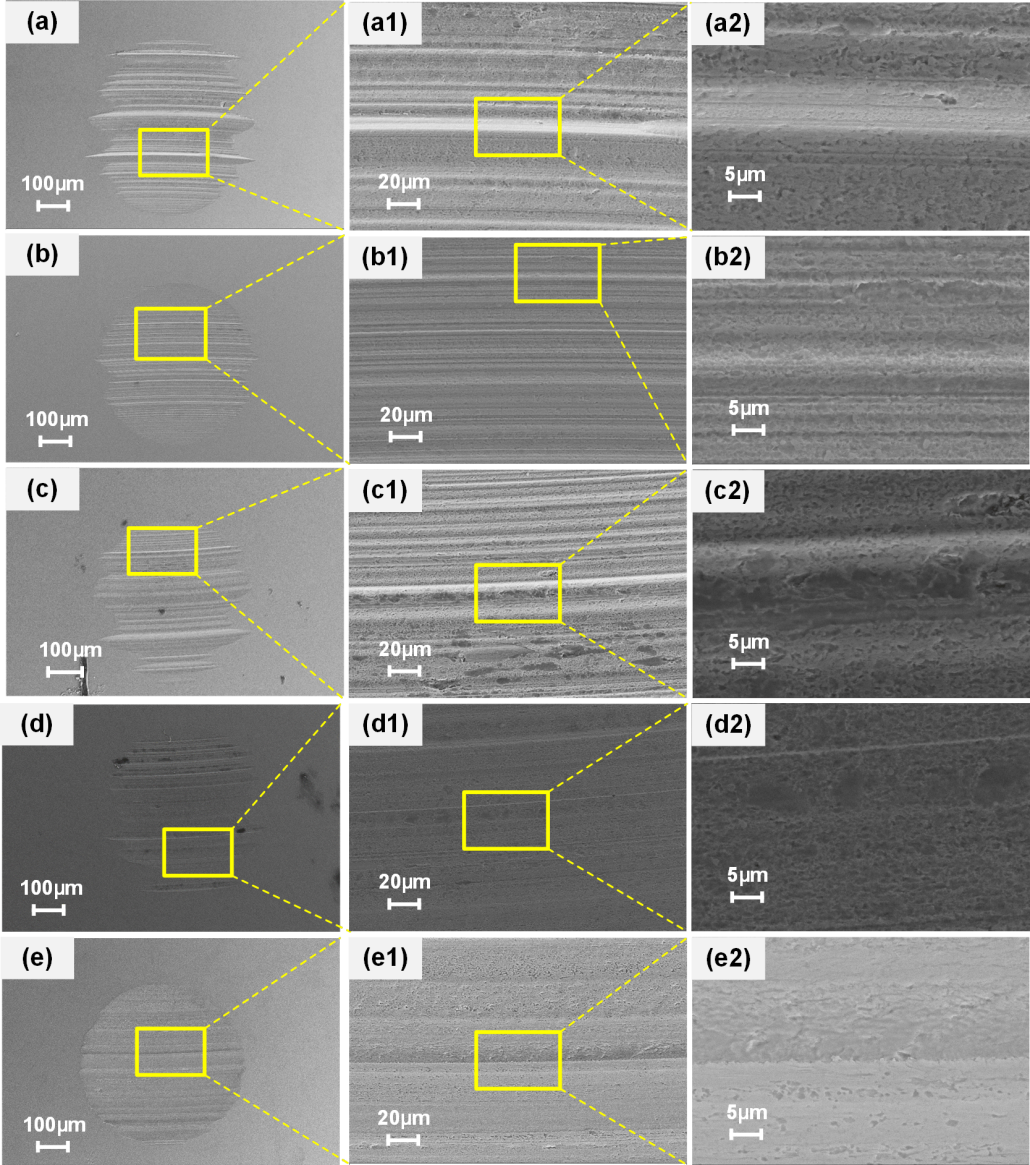
SEM morphologies of worn scar on steel balls lubricated by each grease at 392 N for 60 mins. (a–a2) for Grease G, (b–b2) for Grease G-MA, (c–c2) for Grease G-MB, (d–d2) for Grease G-MC, (e–e2) for Grease G-MX.

Fig 6(a–a2), (b–b2), (c–c2), (d–d2), and (e–e2) present the wear scar morphologies corresponding to Grease G, G-MA, G-MB, G-MC, and G-MX, respectively. For Fig 6(a–a2), Fig 6a displays the morphology at 100 × magnification, with Figs (a1) and (a2) showing successively higher magnifications of the yellow-boxed regions in the preceding image. Similarly, Figs (b–b2), (c–c2), (d–d2), and (e–e2) are arranged in the same manner.

The wear surfaces lubricated by base grease (Grease G) showed greater diameters, a rough edge deeper (Fig 6a), wider furrows, obvious scratches (Fig 6a1 and a2), indicating abrasive wear. In contrast, the wear scars lubricated by each modified grease (Grease G-MA, G-MB, G-MC, and G-MX) displayed smaller diameters (Fig 6b–e), shallower and narrower scratches (Fig 6b1–e2), and less abrasive wear. It was demonstrated that N-G, GN, and PB additives improved the anti-wear properties of titanium complex grease. However, compared with the single-modified greases, the Synergistic-grease (Grease G-MX) led to the smallest diameters (Fig e), the smoothest morphologies and lest furrows (Fig e1, e2), indicating the most excellent anti-wear properties.

Table 8 summarizes EDS compositional analysis along wear track area scans. All tested formulations (Grease G, G-MA, G-MB) exhibited characteristic steel substrate elements (Fe, Cr) and lubricant-derived constituents (C, O, Ti). Notably, carbon atomic concentration followed the hierarchy: G-MC (lowest) < G < G-MX < G-MB < G-MA, correlating directly with N-G/GN additive loading levels. Crucially, only PB-containing formulations (G-MC, G-MX) displayed boron and potassium signatures, confirming residual PB particles within wear tracks through elemental fingerprinting.

**Table 8:** Atomic concentration of typical elements on the worn surface.

Grease	At %						
	CK	FeK	OK	TiK	BK	KK	Cr
G	21.18	61.91	13.73	1.73	0	0	1.45
G-MA	23.02	61.23	13.11	1.23	0	0	1.41
G-MB	22.51	61.78	13.06	1.27	0	0	1.38
G-MC	21.02	62.22	11.44	1.25	1.23	1.49	1.35
G-MX	22.03	61.31	11.62	1.17	1.32	1.26	1.29

#### 3.3.2. XPS analysis on the worn surfaces.

**Fig 7:**
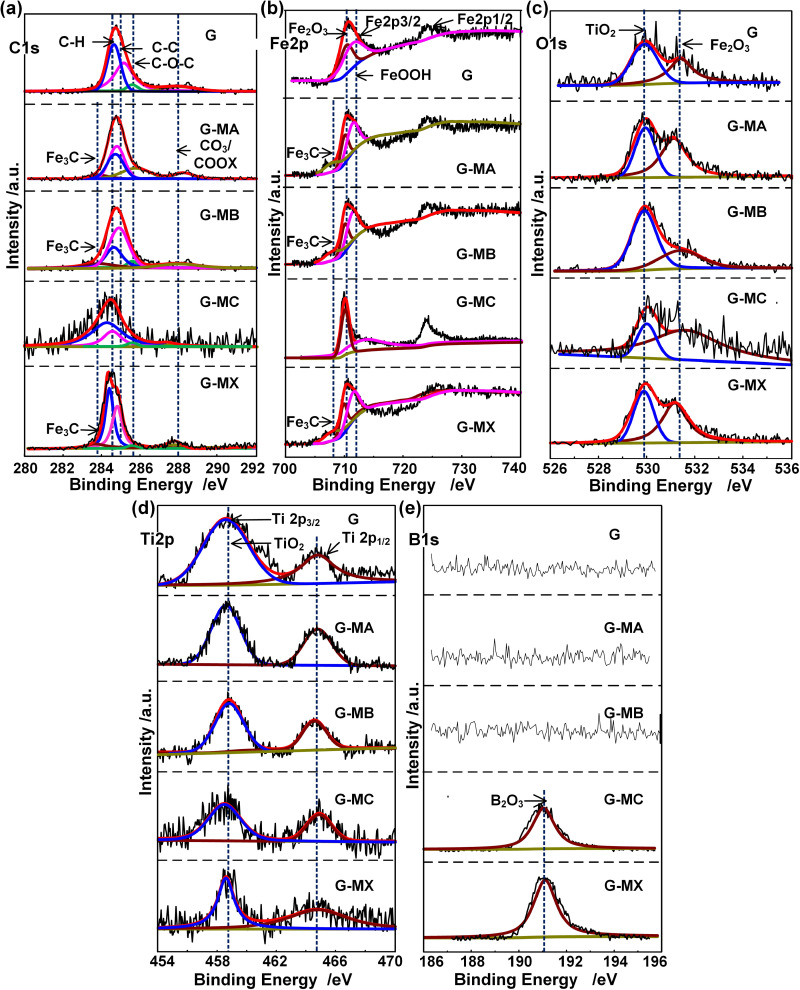
XPS spectra of typical elements on wear scar of steel ball lubricated by each grease at 392 N for 60 mins. (a) Cls, (b) Fe2p, (c) Ols, (d) Ti2p, (e) B1s.

X-ray photoelectron spectroscopy (XPS) analysis was conducted to characterize the chemical states of key elements on wear surfaces. Fig 7 displays high-resolution XPS spectra revealing characteristic binding energy profiles for C 1s, Fe 2p, O 1s, and Ti 2p across all lubricated interfaces. Significantly, B 1s signatures were exclusively detected in PB-modified formulations (G-MC and G-MX), demonstrating selective retention of boron-containing additives within tribochemical films.

XPS deconvolution of the C 1s spectrum (Fig 7a) revealed four characteristic binding energies: 284.6 eV (C-H/C-C bonds), 284.9 eV (graphitic carbon), 285.7 eV (C-O-C ether linkages), and 288.0 eV (carbonate/carboxylate species). This chemical fingerprint confirms titanium complex soap-mediated adsorption-dominated tribofilm formation.

Notably, the 283.9 eV C 1s peak and corresponding 708.1 eV Fe 2p signature (Fig 7a, b) specifically indicated Fe₃C formation in N-G/GN-modified greases (G-MA, G-MB, G-MX). As the primary constituent of white cast iron [50], this iron carbide phase demonstrates inherent wear resistance [51], revealing a triboinduced carburization mechanism where carbon from additives reacts with steel substrates under extreme tribological stress conditions.

XPS analysis revealed significant surface oxidation across all tested formulations. The Fe 2p spectra (Fig 7b) exhibited characteristic Fe³ ⁺ signatures at 710.4 eV (Fe₂O₃) and 712.1 eV, the latter indicating tribochemically formed FeOOH through Fe-COO_x interactions. Concurrent O 1s spectra (Fig 7c) showed dual oxidation evidence: 531.4 eV (Fe₂O₃) and 529.9 eV corresponding to TiO₂ formation, further confirmed by Ti 2p peaks at 458.6 eV (2p₃/₂) and 464.8 eV (2p₁/₂) in Fig 7d. This in situ generated TiO₂ film demonstrates well-documented anti-wear properties through surface passivation [52]. Notably, B 1s spectra (Fig 7e) exclusively exhibited a 191 eV peak (B₂O₃) in PB-modified greases (G-MC/G-MX) [46,47], confirming additive decomposition under extreme tribological conditions. The co-occurrence of TiO₂ and B₂O₃ surface films synergistically enhanced wear resistance through dual mechanochemical protection mechanisms.

## 4. Discussion

The optimized synergistic formulation demonstrated precise additive loading at 0.8307 wt% N-G, 0.0531 wt% GN, and 2.5897 wt% PB. Throughout the applied load range, this formulation achieved superior tribological performance with friction coefficients (AFC: 0.28–0.68) and wear scar diameters (WSD: 360–911 μm) consistently outperforming both base grease and individual additive-modified counterparts. Surface profilometry confirmed exceptional wear track integrity in synergistic grease-lubricated specimens. Mechanistic analysis through XPS revealed tripartite protection mechanisms: (1) additive-derived mechanical reinforcement through particle dispersion, (2) continuous replenishment of physically adsorbed boundary films, and (3) tribochemical film formation via additive-surface interactions, collectively enabling friction/wear mitigation across multiple length scales.

### 4.1. Optimum synergy concentrations of each additive and the prospective applications

Using the compound experiment-Matlab mathematical fitting method, the AFC and WSD functions were developed to determine the optimum synergy concentrations of N-G, GN, and PB, which were 0.8307 wt%, 0.0531 wt%, and 2.5897 wt%, respectively. The optimum synergy combination is not directly chosen from the 15 testing points but from the calculation, which yielded higher accuracy for modifying the grease in tribology.

### 4.2. Lubrication mechanism of synergistically modified titanium complex grease

#### 4.2.1. Physical boundary film.

The boundary film formed on the metal surface serves to protect it against wear. The film respectively originated from the absorbed grease and the physical effects of additives particles.

**Fig 8:**
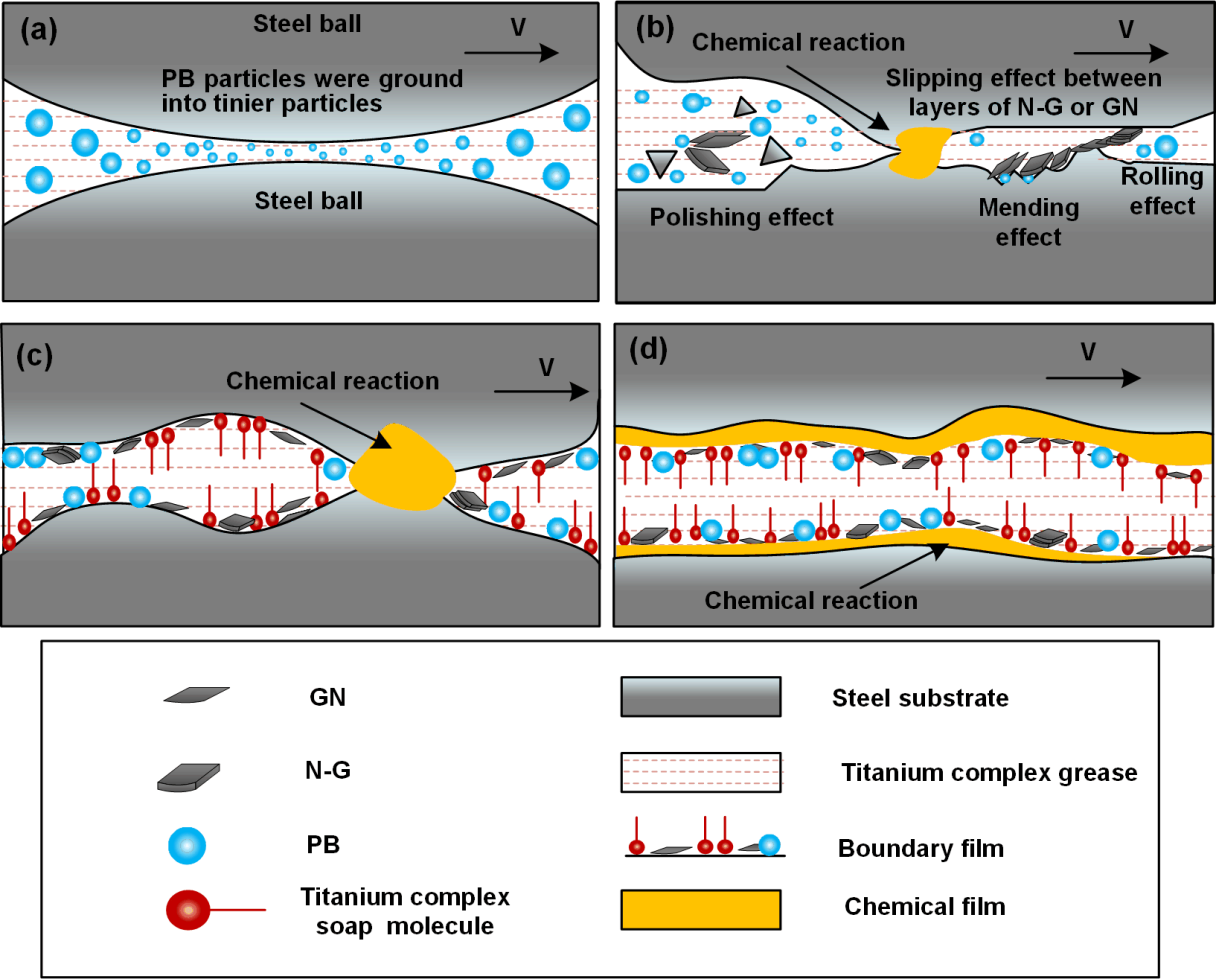
Schematic diagrams of lubricating mechanism for the grease. (a) refinement of potassium borate particles, (b) Physical adsorption of additives and initiation of the tribochemical reaction, (c) Boundary film formation composed of soap molecules and borate particles, with progression of the tribochemical reaction, (d) Stabilized boundary film and formation of the tribochemical reaction film.

For the grease, absorption of molecules of titanium complex soaps occurs in a manner whereby their polar groups anchor onto the metal surface, while the hydrocarbon chains are oriented away from it (shown as Fig 8c), which formed a boundary film [53] to protect the worn steel surface under the lubrication of titanium complex grease. Fig 7a provides evidence for the existence of the boundary film through the presence of C-H, C-C, C-O-C, and CO_3_/COOX groups.

For the additive particles, original microscale PB particles were ground into tinier particles by the rotating steel balls and involved in the frictional interface (shown as Fig 8a). The improved friction reducing and anti-wear properties of the Synergistic-grease were attributed to several factors, including polishing effects, rolling effects, mending effects, and inter-layer sliding effects. For the polishing effect (shown as Fig 8b), refined potassium borate particles, GN and N-G could remove some asperities on the worn surface. For the mending effect (shown as Fig 8b), the ground PB particles, GN and N-G could reduce the roughness of the worn surfaces through filling concave holes. For the rolling effect (shown as Fig 8b), the spherical refined PB particles modified the pure sliding friction to sliding-rolling friction. Sliding effects occurred due to the interlayer sliding of GN of N-G. The polishing effects, mending effects, rolling effects and the sliding effects reduced the AFC of titanium complex grease and improve the friction reducing properties of titanium complex grease. The particles could be absorbed on the worn surfaces, improving the strength of boundary film.

The friction reduction and anti-wear properties improved with increasing additive concentrations due to enhanced strength of the boundary film. However, as shown in Fig 4, further increases in additive concentration resulted in diminishing improvements to these properties. This phenomenon can be attributed to the disruption of the boundary film caused by excessive additive concentrations. Therefore, only at an optimal concentration ratio of the three additives can maximum absorption be achieved, resulting in a boundary film strengthened to its greatest potential and consequently, significantly enhanced friction-reducing and anti-wear properties in the titanium complex grease.

#### 4.2.2. Chemical film.

Among the titanium complex grease, ground PB, GN and N-G and steel substrate chemical reaction occurred under the condition of local high pressure (caused by the loads) and high temperature (caused by high speed friction) (Fig 8b–e), as well as the collision of asperities on worn steel surface. Worn surface underwent elastic and plastic deformation due to the collision between oil from lubricating grease and the asperities under local high pressure. Thereby, the increase of temperature and plastic deformation might increase the diffusion of active atoms from lubrication compounds onto worn surface layer. Consequently, intricate chemical compounds involving iron (Fig 7b), carbon (Fig 7a), oxygen (Fig 7d), titanium (Fig 7c), and boron (Fig 7e) from the lubrication additive and thickener were generated on the worn surface (Fig 8d). The local high temperature and friction caused the breakdown of titanium complex soap molecules, resulting in the formation and deposition of TiO₂ on the worn surface (see Fig 7d). This process enhanced the anti-wear performance of the titanium complex grease. The PB underwent a dual transformation process: firstly, it decomposed into B2O3 which deposited onto the worn surfaces; simultaneously, FeB was generated in situ through a chemical reaction between boron atoms from PB and iron atoms from the worn surfaces. As a result, the anti-wear layer of B_2_O_3_ and FeB improved the anti-wear properties of the titanium complex grease. As the same reason, the element C from the N-G and GN additive reacted with the element Fe from the worn surface, the tribochemical reaction can be described by the formula Fe + 3C → Fe_3_C., and this layer is the main component of white cast iron [50] and possesses good wear resistance [51]. Therefore, During the friction process, chemical reactions occurred between molecules of titanium complex soaps, additives and steel substrate, leading to the formation of complex inorganic chemical compounds such as Fe_3_C, Fe_2_O_3_, FeOOH, TiO_2_, and B_2_O_3_, as evidenced in Fig 7. These compounds were formed and deposited on the worn surface, avoiding the direct contact between mental surface, protecting the worn surfaces.

#### 4.2.3. Complementary of the physical boundary film and chemical film.

Crucially, the tribological enhancement cannot be attributed solely to either film type. The following analysis reveals how their interdependence creates system-level improvements.

(1) Physical boundary film characteristics

The physical boundary film primarily originates from physiosorbed base oil components (e.g., grease soaps) and non-reactive additives [54]. During the initial friction stage, these constituents rapidly adsorb onto worn surfaces through van der Waals interactions and electrostatic forces [55], forming a transient protective layer within seconds [56]. However, such physically adsorbed films exhibit low mechanical resilience [57], rendering them susceptible to shear-induced degradation under prolonged sliding. Pressure-dependent studies reveal optimal film stability at lighter loads. This load-sensitive behavior explains their predominant contribution to friction reduction (μ reduction up to 40% before 392 N in Fig 5a) rather than wear protection.

(2) Chemical boundary film formation

In contrast, chemical boundary films evolve through tribochemically activated processes involving additive decomposition (e.g., ZDDP [58]) and substrate oxidation (Fe + O₂ → Fe_2_O_3_[^7] [59]). Although requiring extended induction periods, these films demonstrate superior durability due to covalent bonding with substrates. High contact pressures promote film generation through mechanisms of thermal activation and mechanical activation. The former is explained by local flash temperatures overcoming reaction energy barriers, while the latter is attributed to strain-induced defect sites enhancing precursor adsorption kinetics. Consequently, chemical films dominate anti-wear performance (WSD reduction 22.4% in Fig 5 b after 510 N) under extreme pressure conditions.

This complementary behaviour aligns with the hierarchical lubrication [60], where physical films provide immediate friction mitigation during run-in periods, while chemically bonded films ensure long-term wear protection under severe operating conditions. The transition between these regimes is governed by the competition between adsorption/desorption kinetics and tribochemical activation thresholds.

### 4.3. Potential limitations and further explorations

This study systematically demonstrates the enhanced tribological performance of titanium complex grease synergistically modified with GN, N-G, and PB (0.28–0.68 AFC; 23.3% WSD reduction at 598 N), though two key limitations guide future work: (1) the constant-speed testing protocol (1450 rpm) necessitates expansion to variable velocities to better mimic operational dynamics, and (2) surface texturing effects—critical for advanced lubricant engineering—remain unexplored despite smooth-surface mechanistic revelations of tribochemical films and additive interactions. Industrially, the formulation’s extreme-pressure resilience (WSD 366910 μm under 96–598 N) suits heavy-load applications like mining drivetrains and offshore wind gearboxes, while its eco-friendly potassium borate component aligns with marine corrosion resistance demands. Further research will bridge these scientific and engineering gaps by probing velocity-dependent behavior and optimizing surface texture parameters to develop next-generation lubrication systems balancing performance and sustainability.

## 5. Conclusions

This study systematically investigates the synergistic effects of nano-graphite (N-G), graphene (GN), and potassium borate (PB) additives in enhancing the tribological performance of titanium complex grease. Through an integrated experimental-computational methodology combining tribological testing and MATLAB-based response surface optimization, we identified optimal additive concentrations at 0.8307 wt% N-G, 0.0531 wt% GN, and 2.5897 wt% PB. The resulting synergistic formulation (Grease G-MX) exhibited superior performance compared to single-additive formulations and base grease.

The enhanced lubrication arises from multiscale synergistic mechanisms operating across physical and chemical domains. N-G and GN particles mechanically polish surfaces while facilitating interlayer sliding/rolling, complemented by physisorbed boundary films. Concurrent tribochemical reactions between additives, titanium soap molecules, and steel substrates generate a protective layer comprising Fe₃C, Fe₂O₃, TiO₂, and B₂O₃. This layer prevents direct contact between metal surfaces, thereby protecting the worn surfaces.

## References

[1] Wang J, Zhang H, Hu W, Li J. Tribological Properties and Lubrication Mechanism of Nickel Nanoparticles as an Additive in Lithium Grease. Nanomaterials (Basel). 2022;12(13):2287. doi: 10.3390/nano12132287 35808123 PMC9268507

[2] Razavi S, Sabbaghi S, Rasouli K. Comparative investigation of the influence of CaCO_3_ and SiO_2_ nanoparticles on lithium-based grease: Physical, tribological, and rheological properties. Inorganic Chemistry Communications. 2022;142:109601. doi: 10.1016/j.inoche.2022.109601

[3] Qiang H, Wang T, Qu H, Zhang Y, Li A, Kong L. Tribological and rheological properties of nanorods–Al_2_O_3_ as additives in grease. Proceedings of the Institution of Mechanical Engineers, Part J: Journal of Engineering Tribology. 2018;233(4):605–14. doi: 10.1177/1350650118787403

[4] Mohamed A, Hamdy M, Bayoumi M, Osman T. Synthesis and tribological properties of nanogrease. Ind Lubr Tribol. 2018;70(3):512–8. doi: 10.1108/ilt-08-2017-0228

[5] Qiao J, Chen Z, Zhao J, Ren J, Wang H, Zhi C, et al. Graphene promotes the growth of Vigna angularis by regulating the nitrogen metabolism and photosynthesis. PLoS One. 2024;19(3):e0297892. doi: 10.1371/journal.pone.0297892 38451974 PMC10919591

[6] Zhao J, Mao J, Li Y, He Y, Luo J. Friction-induced nano-structural evolution of graphene as a lubrication additive. Applied Surface Science. 2018;434:21–7. doi: 10.1016/j.apsusc.2017.10.119

[7] Tang Z, Li S. A review of recent developments of friction modifiers for liquid lubricants (2007–present). Current Opinion in Solid State and Materials Science. 2014;18(3):119–39. doi: 10.1016/j.cossms.2014.02.002

[8] Mura A, Curà F, Adamo F. Evaluation of graphene grease compound as lubricant for spline couplings. Tribology International. 2018;117:162–7. doi: 10.1016/j.triboint.2017.08.027

[9] Kumar P, Wani MF. Synthesis and tribological properties of graphene: A review. J Tribologi. 2017;13:36–71.

[10] Ni J, Feng G, Meng Z, Hong T, Chen Y, Zheng X. Reinforced lubrication of vegetable oils with graphene additive in tapping ADC12 aluminum alloy. Int J Adv Manuf Technol. 2017;94(1–4):1031–40. doi: 10.1007/s00170-017-0952-3

[11] Guo Y-B, Zhang S-W. The Tribological Properties of Multi-Layered Graphene as Additives of PAO2 Oil in Steel–Steel Contacts. Lubricants. 2016;4(3):30. doi: 10.3390/lubricants4030030

[12] Jing W, Xiaochuan G, Yan H, Mingjun J, Wanqing G, Yuantao Z, et al. Tribological characteristics of graphene as lithium grease additive. China Pet Process PetrochemTechnol 2017;19(1):46–54.

[13] Kamel BM, Mohamed A, El Sherbiny M, Abed KA, Abd-Rabou M. Tribological properties of graphene nanosheets as an additive in calcium grease. Journal of Dispersion Science and Technology. 2016;38(10):1495–500. doi: 10.1080/01932691.2016.1257390

[14] Farhanah Azman N, Samion S, Paiman Z, Kameil Abdul Hamid M. Tribological performance and mechanism of graphite, hBN and MoS2 as nano-additives in palm kernel oil-based lubricants: A comparative study. Journal of Molecular Liquids. 2024;410:125616. doi: 10.1016/j.molliq.2024.125616

[15] Su Y, Gong L, Chen D. An Investigation on Tribological Properties and Lubrication Mechanism of Graphite Nanoparticles as Vegetable Based Oil Additive. Journal of Nanomaterials. 2015;2015(1). doi: 10.1155/2015/276753

[16] Vidal FAC, Ávila AF. Tribological Investigation of Nanographite Platelets as Additive in Anti-Wear Lubricant: A Top-Down Approach. Journal of Tribology. 2014;136(3). doi: 10.1115/1.4027479

[17] Huang HD, Tu JP, Gan LP, Li CZ. An investigation on tribological properties of graphite nanosheets as oil additive. Wear. 2006;261(2):140–4. doi: 10.1016/j.wear.2005.09.010

[18] Zhang Z, Cai Z, Peng J, Zhu M. Comparison of the tribology performance of nano-diesel soot and graphite particles as lubricant additives. J Phys D: Appl Phys. 2015;49(4):045304. doi: 10.1088/0022-3727/49/4/045304

[19] Shahira Liza Kamis BIY, Kanao F, Noor Ayuma Mat T, Ikmal A, Ahmad Sukri MAA. Tribological properties of graphite particles as an anti-friction and anti-wear additive in lithium soap grease. Jurnal Tribologi. 2024;40(2024):179–98.

[20] Kumar N, Saini V, Bijwe J. Tribological investigations of nano and micro-sized graphite particles as an additive in lithium-based grease. Tribol Lett. 2020;68(4):1–4. doi: 10.1007/s11249-020-01362-1

[21] Kong L, Hu H, Wang T, Huang D, Fu J. Synthesis and surface modification of the nanoscale cerium borate as lubricant additive. Journal of Rare Earths. 2011;29(11):1095–9. doi: 10.1016/s1002-0721(10)60605-9

[22] Boshui C, Kecheng G, Jianhua F, Jiang W, Jiu W, Nan Z. Tribological characteristics of monodispersed cerium borate nanospheres in biodegradable rapeseed oil lubricant. Applied Surface Science. 2015;353:326–32. doi: 10.1016/j.apsusc.2015.06.107

[23] Hu ZS, Lai R, Lou F, Wang LG, Chen ZL, Chen GX, et al. Preparation and tribological properties of nanometer magnesium borate as lubricating oil additive. Wear. 2002;252(5–6):370–4. doi: 10.1016/s0043-1648(01)00862-6

[24] Hu ZS, Dong JX, Chen GX, He JZ. Preparation and tribological properties of nanoparticle lanthanum borate. Wear. 2000;243(1–2):43–7. doi: 10.1016/s0043-1648(00)00415-4

[25] Li J, Hao L, Xu X, Ren T. Tribological synergism of surface‐modified calcium borate nanoparticles and sulfurized olefin. Industrial Lubrication and Tribology. 2012;64(4):217–23. doi: 10.1108/00368791211232762

[26] Han S, Liu S, Wang Y, Zhou X, Hao L. Preparation, Characterization, and Tribological Evaluation of a Calcium Borate Embedded in an Oleic Acid Matrix. Ind Eng Chem Res. 2012;51(43):13869–74. doi: 10.1021/ie300940r

[27] Normand V, Martin JM, Ponsonnet L, Inoue K. Tribology Letters. 1998;5(2/3):235–42. doi: 10.1023/a:1019129305504

[28] Liu N, Tian Y, Yu L, Li Q, Meng F, Zheng Y, et al. Synthesis and surface modification of uniform barium borate nanorods for lubrication. Journal of Alloys and Compounds. 2008;466(1–2):L11–4. doi: 10.1016/j.jallcom.2007.11.053

[29] Hu ZS, Dong JX. Study on antiwear and reducing friction additive of nanometer titanium borate. Wear. 1998;216(1):87–91. doi: 10.1016/s0043-1648(97)00249-4

[30] Savrık SA, Balköse D, Ülkü S. Synthesis of zinc borate by inverse emulsion technique for lubrication. J Therm Anal Calorim. 2010;104(2):605–12. doi: 10.1007/s10973-010-1159-0

[31] Li Y, Zhang S, Ding Q, Tang J, Qin B, Hu L. The extreme pressure and lubricating behaviors of potassium borate nanoparticles as additive in PAO. Particulate Science and Technology. 2018;37(8):932–42. doi: 10.1080/02726351.2018.1458353

[32] Li H, Zeng F, Yin Z, Jiang D, Huo Y. A study on the tribological behavior of hybrid PTFE/Kevlar fabric composites filled with nano‐SiC and/or submicron‐WS2 fillers. Polymer Composites. 2015;37(7):2218–26. doi: 10.1002/pc.23400

[33] Qian S, Wang H, Huang C, Zhao Y. Experimental investigation on the tribological properties of modified carbon nanotubes as the additive in castor oil. Ind Lubr Tribol. 2018;70(3):499–505. doi: 10.1108/ilt-05-2017-0138

[34] Shi X, Liskiewicz TW, Beake BD, Chen J, Wang C. Tribological performance of graphite-like carbon films with varied thickness. Tribology International. 2020;149:105586. doi: 10.1016/j.triboint.2019.01.045

[35] Abhinav M, Manoj A, Satardekar P, Saravanan P, Narala SKR. Effect of solid lubricant particles on the tribological behavior of grease. Jurnal Tribologi 2024;40(2024):247–67.

[36] Nassef MGA, Nassef BG, Hassan HS, Nassef GA, Elkady M, Pape F. Tribological and Chemical–Physical Behavior of a Novel Palm Grease Blended with Zinc Oxide and Reduced Graphene Oxide Nano-Additives. Lubricants. 2024;12(6):191. doi: 10.3390/lubricants12060191

[37] Xin YL, Hu JQ, Yang SZ. Extreme pressure synergistic properties and tribological behaviors of several additives in lithium greases. Surface Technology. 2017;46(07):97–103. [In Chinese].

[38] Varalakshmi M, Venugopal Reddy V. An Experimental Investigation on Tribological Behaviour of Polyalphaolefin (PAO4) Oil Modified with Cu/MnS Nanocomposites. Proceedings of the Institution of Mechanical Engineers, Part J: Journal of Engineering Tribology. 2022;237(4):954–63. doi: 10.1177/13506501221140448

[39] Wu C, Li S, Ni J, Yao L, Xia Q. Effect of structure of ZnO and SiO_2_ core-shell composite nanoparticles as lubricant additive on tribological properties of greases. Appl Surf Sci. 2024;657:159745. doi: 10.1016/j.apsusc.2024.159745

[40] Wang Y, Zhang P, Gao X, Cheng Y. Rheological and tribological properties of polyurea greases containing additives of MoDDP and PB. Tribology International. 2023;180:108291. doi: 10.1016/j.triboint.2023.108291

[41] Shen T, Wang D, Yun J, Liu Q, Liu X, Peng Z. Tribological properties and tribochemical analysis of nano-cerium oxide and sulfurized isobutene in titanium complex grease. Tribology International. 2016;93:332–46. doi: 10.1016/j.triboint.2015.09.028

[42] Nagarajan T, Sridewi N, Wong WP, Walvekar R, Khanna V, Khalid M. Synergistic performance evaluation of MoS_2_-hBN hybrid nanoparticles as a tribological additive in diesel-based engine oil. Sci Rep. 2023;13(1):12559. Epub 2023/08/03. doi: 10.1038/s41598-023-39216-0 ; PMCID: PMC10397337 37532805 PMC10397337

[43] Alghani W, Ab Karim MS, Bagheri S, Amran NAM, Gulzar M. Enhancing the Tribological Behavior of Lubricating Oil by Adding TiO_2_, Graphene, and TiO_2_/Graphene Nanoparticles. Tribology Transactions. 2019;62(3):452–63. doi: 10.1080/10402004.2019.1573282

[44] Abdullah MIHC, Abdollah MFB, Amiruddin H, Tamaldin N, Nuri NRM. Optimization of Tribological Performance of hBN/AL_2_O_3_ Nanoparticles as Engine Oil Additives. Procedia Engineering. 2013;68:313–9. doi: 10.1016/j.proeng.2013.12.185

[45] Hou X, Tang H, Dai L, Li X, Lan G, Ai Z, et al. Potassium borate/graphene nanocomposite lubricant additive with anti-friction/wear and anti-corrosion functions for marine diesel engine burning low sulfur fuel. Wear. 2024;550–551:205395. doi: 10.1016/j.wear.2024.205395

[46] Yan J, Zeng H, Liu T, Mai J, Ji H. Tribological Performance and Surface Analysis of a Borate Calcium as Additive in Lithium and Polyurea Greases. Tribology Transactions. 2016;60(4):621–8. doi: 10.1080/10402004.2016.1194506

[47] Yang Y, Wang X, Mei S, Zhu X, Chen S, Xiong P, et al. Preparation and tribological properties of BN/calcium borate nanocomposites as additive in lubricating oil. Ind Lubr Tribol. 2018;70(1):105–14. doi: 10.1108/ilt-10-2016-0255

[48] Myers RH, Montgomery DC, Anderson-Cook CM. Response surface methodology: process and product optimization using designed experiments. John Wiley & Sons; 2016.

[49] Singh J, Kumar D, Tandon N. Development of Nanocomposite Grease: Microstructure, Flow, and Tribological Studies. Journal of Tribology. 2017;139(5). doi: 10.1115/1.4035775

[50] Tsuzuki A, Sago S, Hirano S-I, Naka S. High temperature and pressure preparation and properties of iron carbides Fe7C3 and Fe3C. J Mater Sci. 1984;19(8):2513–8. doi: 10.1007/bf00550805

[51] Laird G, Powell GLF. Solidification and solid-state transformation mechanisms in Si alloyed high-chromium white cast irons. Metall Trans A. 1993;24(4):981–8. doi: 10.1007/bf02656520

[52] Vera ML, Rosenberger MR, Schvezov CE, Ares AE. Wear Resistance of Anodic Titanium Dioxide Films Produced on Ti-6Al-4V Alloy. Nanomaterials and Nanotechnology. 2015;5:6. doi: 10.5772/60069

[53] Adhvaryu A, Sung C, Erhan SZ. Fatty acids and antioxidant effects on grease microstructures. Industrial Crops and Products. 2005;21(3):285–91. doi: 10.1016/j.indcrop.2004.03.003

[54] Spikes H. Friction Modifier Additives. Tribol Lett. 2015;60(1). doi: 10.1007/s11249-015-0589-z

[55] Campen S, Green JH, Lamb GD, Spikes HA. In Situ Study of Model Organic Friction Modifiers Using Liquid Cell AFM; Saturated and Mono-unsaturated Carboxylic Acids. Tribol Lett. 2015;57(2). doi: 10.1007/s11249-015-0465-x

[56] Gosvami NN, Bares JA, Mangolini F, Konicek AR, Yablon DG, Carpick RW. Tribology. Mechanisms of antiwear tribofilm growth revealed in situ by single-asperity sliding contacts. Science. 2015;348(6230):102–6. doi: 10.1126/science.1258788 25765069

[57] Sawyer WG, Argibay N, Burris DL, Krick BA. Mechanistic Studies in Friction and Wear of Bulk Materials. Annu Rev Mater Res. 2014;44(1):395–427. doi: 10.1146/annurev-matsci-070813-113533

[58] Neville A, Morina A, Haque T, Voong M. Compatibility between tribological surfaces and lubricant additives—How friction and wear reduction can be controlled by surface/lube synergies. Tribology International. 2007;40(10–12):1680–95. doi: 10.1016/j.triboint.2007.01.019

[59] Morina A, Neville A, Priest M, Green JH. ZDDP and MoDTC interactions in boundary lubrication—The effect of temperature and ZDDP/MoDTC ratio. Tribology International. 2006;39(12):1545–57. doi: 10.1016/j.triboint.2006.03.001

[60] Zhou X, Qiu S, Liu L, Xing W, He L, Hou Y, et al. Hierarchical hollow SiO_2_@TiO_2_ sphere structure for enhancing the lubrication and photo-catalytic degradation of liquid paraffin. Composites Part B: Engineering. 2019;167:599–607. doi: 10.1016/j.compositesb.2019.03.019

